# Exploring Electrospun Scaffold Innovations in Cardiovascular Therapy: A Review of Electrospinning in Cardiovascular Disease

**DOI:** 10.3390/bioengineering11030218

**Published:** 2024-02-25

**Authors:** Mark Broadwin, Frances Imarhia, Amy Oh, Christopher R. Stone, Frank W. Sellke, Sankha Bhowmick, M. Ruhul Abid

**Affiliations:** 1 Division of Cardiothoracic Surgery, Department of Surgery, Cardiovascular Research Center, Rhode Island Hospital, Alpert Medical School of Brown University, Providence, RI 02903, USA; mbroadwin@lifespan.org (M.B.); frances_imarhia@brown.edu (F.I.); hyunju_oh@brown.edu (A.O.); christopher_stone@brown.edu (C.R.S.); frank_sellke@brown.edu (F.W.S.); 2 Department of Mechanical Engineering, University of Massachusetts Dartmouth, North Dartmouth, MA 02747, USA; sbhowmick@umassd.edu

**Keywords:** electrospinning, cardiovascular disease, nanofiber, biocompatible, drug delivery, scaffold

## Abstract

Cardiovascular disease (CVD) remains the leading cause of mortality worldwide. In particular, patients who suffer from ischemic heart disease (IHD) that is not amenable to surgical or percutaneous revascularization techniques have limited treatment options. Furthermore, after revascularization is successfully implemented, there are a number of pathophysiological changes to the myocardium, including but not limited to ischemia-reperfusion injury, necrosis, altered inflammation, tissue remodeling, and dyskinetic wall motion. Electrospinning, a nanofiber scaffold fabrication technique, has recently emerged as an attractive option as a potential therapeutic platform for the treatment of cardiovascular disease. Electrospun scaffolds made of biocompatible materials have the ability to mimic the native extracellular matrix and are compatible with drug delivery. These inherent properties, combined with ease of customization and a low cost of production, have made electrospun scaffolds an active area of research for the treatment of cardiovascular disease. In this review, we aim to discuss the current state of electrospinning from the fundamentals of scaffold creation to the current role of electrospun materials as both bioengineered extracellular matrices and drug delivery vehicles in the treatment of CVD, with a special emphasis on the potential clinical applications in myocardial ischemia.

## 1. Introduction

Ischemic heart disease (IHD) is responsible for over 16% of all-cause mortality, according to the World Health Organization, constituting it as the leading cause of death worldwide [1]. Although the mainstay of treatment for IHD remains revascularization, there are many patients who are not amenable to surgical or percutaneous revascularization intervention and for whom treatment options therefore remain limited. Furthermore, even after revascularization is successfully implemented, there are many pathophysiological changes that occur in the myocardium, including ischemia-reperfusion injury, necrosis, altered inflammation, tissue remodeling, and dyskinetic wall motion; while these changes are well-characterized, there are currently limited effective treatment options available to address them [2].

Multiple treatment strategies have attempted to address this dearth of options, ranging from pharmacologic interventions and gene therapy to biomedical device development. Unfortunately, IHD has exhibited resistance to many of these therapies. In the case of pharmacologic intervention, this is likely due to the nature of the disease process: as the area of injury is characterized by compromised blood flow, delivery of a pharmaceutical compound to the affected region is correspondingly compromised. Moreover, drugs given systemically may induce undesired side effects. Circumventing this problem with bioengineered substances appears to be an attractive option in this regard because this strategy allows for a more specifically targeted approach, but this is often limited by the method of delivery and the anatomy of the targeted myocardium. Recently, electrospinning scaffolds using biocompatible biopolymers have become an area of interest, as this technology allows for the combination of both bioengineered structural support as well as localized, measured, and sustained pharmacologic delivery [3].

Electrospinning is a process by which a nanofiber scaffold is created by subjecting a polymer solution to a high-voltage field. Due to the heterogeneity of substances able to undergo this process, the properties of the scaffolds can be precisely countertrolled and offer an opportunity to tailor the scaffold structure to desired parameters for optimized integration into the target anatomy [4]. Furthermore, electrospun scaffolds can be generated from myriad solutions with dissolved bioactive compounds, allowing for regional drug delivery to areas of interest without the need for systemic administration and bypassing the circulatory system as a mechanism for drug delivery [5].

Despite the promise of this technology, however, the role of electrospun scaffolds in the treatment of cardiovascular disease remains in its infancy. In this review, we aim to discuss the current state of electrospinning from the fundamentals of scaffold creation to the current role of electrospun materials as both bioengineered extracellular matrices and drug delivery vehicles in the treatment of cardiac pathophysiology, as well as the potential clinical applications of this technology.

## 2. Fundamentals of Electrospinning

### 2.1. Definition and Principles of Electrospinning

The first patent for electrospinning was filed by Frances Cooley in 1900, but it was not until the work of Anton Formhals in the 1930s that electrospinning began to resemble the process we use today. Formhals filed 22 patents related to electrospinning that involved advances in charged spinners, nozzle design, and alteration in current flow. These advances marked the beginning of the popularity of this technique [6,7]. Electrospinning is a versatile and precise nanofiber fabrication technique widely employed in materials science, nanotechnology, and various other scientific domains. This process entails the creation of ultrafine polymer or composite fibers, with diameters typically ranging from micrometers to nanometers. It is achieved by subjecting a polymer solution to a high-voltage electric field. The simplicity and practicality of this technique render it highly appealing for applications in the field of bioengineering.

### 2.2. Electrospinning Process and Parameters

**Figure 1:**
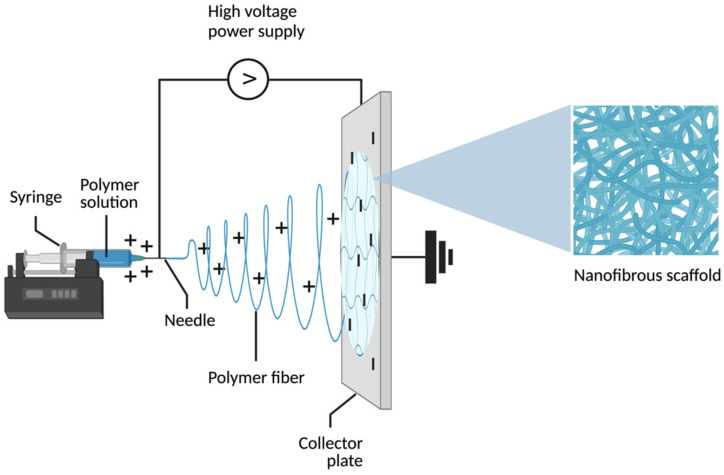
Schematic diagram of the basic elements required in the manufacturing of electrospun nanoscaffolds. Polymer solutions are subjected to a high-voltage field and extruded from a nozzle, fracturing into identical nanofiber bands as they are collected on a grounded collector plate.

Although there are various methods of electrospinning, the electrospinning process generally relies on three essential components: a syringe with a nozzle tip connected to a high-voltage direct current (HVDC) source, a flow rate regulator, and a grounded collecting screen that can be assembled in a variety of different ways [4] (Figure 1). The HVDC source generates an electric current, inducing the extrusion of a positively charged liquid jet composed of a polymer solution, melt, or emulsion from the nozzle tip. As this jet stream extends, it spontaneously fragments into numerous identical randomly oriented fibers, which are subsequently collected on the negative grounded collecting screen. The outcome is a fibrous scaffold, highly esteemed in the realm of tissue engineering for its capacity to mimic the native structure of the extracellular matrix and enhance cellular activities due to the resulting large surface area-to-volume ratio [7].

**Figure 2:**
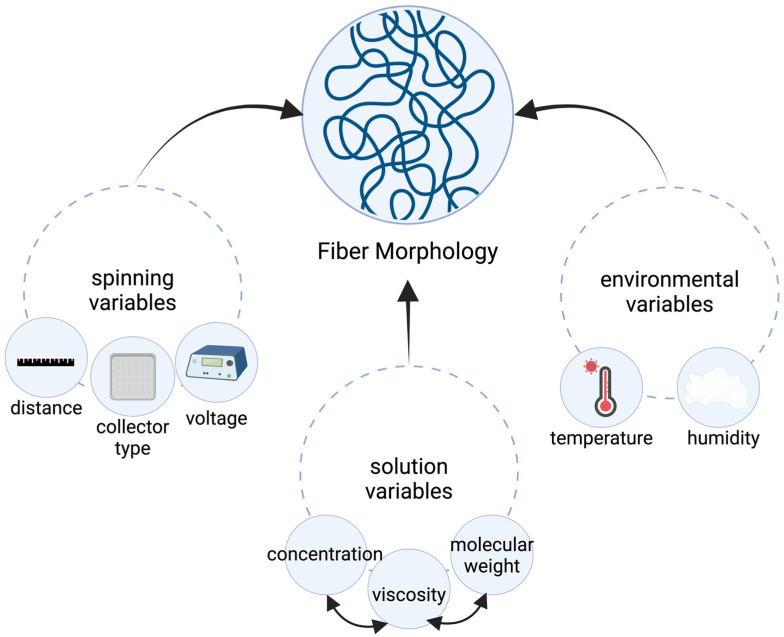
Nanofiber structure is influenced broadly by three categories of variables: environmental variables, solution variables, and spinning variables. Environmental variables include temperature and humidity. Solution variables include solution concentration, viscosity, and molecular weight. Spinning variables include voltage, collector type, and distance of nozzle to collector.

An additional benefit of the electrospinning process is the number of different ways it can be manipulated to alter various characteristics of the fibrous scaffold. These parameters are often classified in terms of solution properties, controlled variables, and ambient parameters (Table 1). Solution properties include the solution viscosity, surface tension, polymer molecular weight, and conductivity. Controlled variables in this process encompass the regulation of parameters like flow rate, electric field strength, the separation distance between the nozzle tip and collector, needle tip design, and the composition and geometry of the collector. Furthermore, ambient conditions, including temperature, humidity, and air velocity, also play a crucial role in this context (Figure 2). These parameters have been extensively studied in reviews by Chinnappan et al., Pham et al., and Bhattarai et al. [4,8,9].

Of the solution properties, one of the biggest determiners of fiber size and morphology is the solution viscosity. A polymeric solution requires an adequate viscosity to overcome surface tension to yield continuous fibers without beading. Because a polymeric solution’s viscosity is closely related to its concentration, molecular weight, and surface tension, the effects of modifying these parameters are often tightly linked [4]. Based on studies performed on a wide variety of polymers, it was observed that imperfections in the form of beading and droplets can occur at low polymer solution concentrations due to the initiation of electrospraying rather than electrospinning [4,10,11,12,13]. On the other hand, exceptionally high concentrations are associated with the production of non-nanoscaled and helix-shaped microribbons rather than the intended smooth nanofibers [4,8,12]. Similarly, an increase in the polymer solution’s molecular weight has been shown to cause an increase in fiber diameter and corresponding alterations in fiber morphology with higher molecular weights associated with the formation of smoother nanofibers [14]. Modifications to the solution’s surface tension can be induced by altering the solvent used in the solution. In some cases, various solvents, including ethanol, have been noted to cause a reduction in the surface tension with a resulting decrease in beading. It is also noted that enhancing the polymer solution’s conductivity or charge density can result in the production of more consistent fibers with a reduced occurrence of beads [4,8]. This can be attributed to the stronger tensile forces exhibited by highly conductive solutions. In most cases, this modification is also accompanied by a decrease in fiber diameter [15].

Studies on the effect of the polymer solution’s flow rate through the syringe have shown that high flow rates produce thick-diameter bead fibers rather than the smooth filaments produced at low flow rates. This is thought to be due to insufficient drying times associated with high flow rates [4,8,9,16]. The effects of the electric field strength, as determined by the voltage supplied to the electrospinning apparatus nozzle, are less straightforward. When studying the effects of varying applied voltage during electrospinning on the properties of polystyrene (PS) fibers, a drop in the size of PS fibers from approximately 20 to 10 μm was noted as the spinning voltage increased from 5 to 12 kV. Interestingly, keeping all other conditions constant, beading defects were observed in the fibers only at the extreme voltages of 5 and 12 kV [16]. However, a different study on the electrospinning of polysulfone (PSf) nanofibers revealed that applied voltage significantly influenced fiber diameter, with an initial increase up to 15 kV followed by a decrease in diameter as voltage continued to rise [17]. This relationship seems to be inconsistent across different polymer formulations [8]. The distance between the syringe tip and collector also plays a crucial role in determining the diameter and structure of electrospun fibers. If the distance is too short, the fibers may not solidify before reaching the collector, and if it is too long, bead-like fibers can form [4,8]. Therefore, the separation distance must be optimized on a case-by-case basis.

Ambient conditions are an understated determinant of fiber size and morphology. Several studies have shown that increasing the temperature of a polymer solution results in the production of fibers with a smaller diameter. When studying the impact of solution temperature on electrospun polyamide-6 (PA-6) fibers, it was found that the average fiber diameter decreased from approximately 98 nm at 30 °C to around 90 nm at 60 °C. This change in morphology was attributed to the associated decrease in the solution’s viscosity [18]. When studying relative humidity effects on over 20 hydrophobic and hydrophilic polymers, a study conducted by Szewczyk and Stachewicz found that relative humidity stands out as one of the most critical parameters in the electrospinning process. Low relative humidity was noted to lead to charge buildup and fiber defects, while high relative humidity caused charge dissipation, impacting fiber formation and leading to varied fiber diameters and morphologies. This variability is particularly evident in hydrophobic polymers, while hydrophilic polymers are less affected. Overall, humidity exerts a profound influence on nearly all aspects of the resulting fibers, encompassing crystallinity, mechanical properties, morphology, wetting characteristics, mesh density, and fiber diameter [19].

While it may be challenging to isolate certain parameters owing to their interdependence, it remains crucial to assess the impact of each parameter on the morphology of electrospun fibers. The versatility inherent in the electrospinning process represents one of its most valuable aspects, with these parameters playing a pivotal role in this regard.

### 2.3. Selection of Polymers and Nanofibers in Cardiac Tissue Engineering

Tissue engineering is an emerging interdisciplinary field that combines principles from engineering and life sciences to achieve tissue regeneration. This approach utilizes cells, biomaterials, or a combination of both to support, mend, or enhance tissue function. Biomaterials play a crucial role in tissue engineering by serving as scaffolds that support` cellular growth and adhesion. The main aim of scaffold production in tissue engineering is to replicate the natural extracellular matrix on a nanoscale to create a conducive environment for cell growth and attachment. To achieve this goal, innovative methods have been devised for crafting three-dimensional scaffolds using biomaterials [20].

Polymers offer considerable processing versatility and are highly favored as scaffolding biomaterials due to their biocompatibility and biodegradability. Within this category, a range of natural polymers such as chitosan, gelatin, collagen, and alginate, along with synthetic polymers including polylactide (PLA), poly(lactic-co-glycolic acid) (PLGA), polycaprolactone (PCL), poly(glycerol sebacate), and polyurethane (PU), stand out as prominent choices for scaffolds in tissue engineering. While natural materials tend to have a low immunogenicity, they are often limited in their mechanical properties. On the other hand, synthetic polymers have highly controllable mechanical properties but can be more likely to induce an immune response [21] (Table 2). Contemporary approaches to cardiac tissue engineering may incorporate nanofibers and polymers, typically in conjunction with electrical stimulation, stem cell therapy, or drug release, aimed at enhancing the functionality and regeneration of cardiac tissue, as demonstrated in animal studies [21,22,23,24].

## 3. Electrospinning in Cardiac Tissue Engineering

### 3.1. Electrospun Nanofibers for Myocardial Regeneration

#### 3.1.1. Enhancing Cell Adhesion and Proliferation

**Figure 3:**
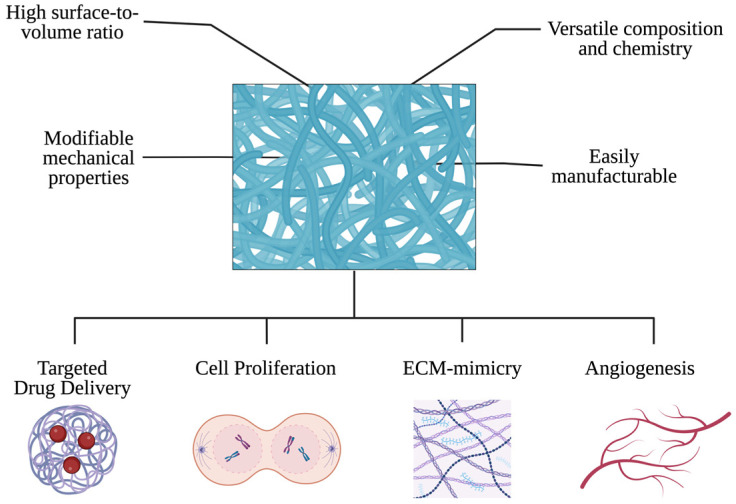
Schematic diagram of some of the demonstrated properties of electrospun scaffolds that may be leveraged for the treatment of cardiovascular disease. The ability of electrospun nanoscaffolds to mimic native tissue extracellular matrix (ECM) makes them highly attractive bioengineered materials. This ECM mimicry, in turn, facilitates creation of a microenvironment that is conducive to both cellular proliferation and angiogenesis. Furthermore, the high surface-to-volume ratio makes electrospun scaffolds ideal vehicles for sustained, timed, and targeted drug delivery.

In recent years, electrospun scaffolds have garnered immense popularity in the field of cardiac tissue engineering. To ensure the proper functioning of tissues and organs, scaffolds must be designed to facilitate direct cell integration into three-dimensional structures and support tissue regeneration. Various manufacturing techniques have been explored to create synthetic porous graft scaffolds, with electrospinning emerging as a novel method for scaffold fabrication. This technique can produce interconnected meshes comprising fibers ranging from a few nanometers to several micrometers in diameter, closely resembling the extracellular matrix of native tissue. This fabrication method has been proven to result in enhanced cell adhesion and proliferation in various studies (Figure 3).

When evaluating and comparing the physical and biocompatible attributes of electrospun PCL, PLGA, and coaxial scaffolds, a study by Bazgir et al. revealed that all three biodegradable polymeric scaffolds efficiently supported cell adhesion, proliferation, and viability for both human umbilical vein endothelial cells and human vascular fibroblasts. Particularly noteworthy was the superior adhesion and markedly increased cell proliferation observed in PLGA membranes when compared to a control group without polymers [53]. A similar study also utilized electrospinning to develop a scaffold to optimize cell adhesion, biocompatibility, osteogenic, and antibacterial properties. This study developed a novel PLGA/PCL electrospun scaffold named PP-pDA-Ag-COL, which incorporated silver nanoparticles (AgNPs) using a mussel-inspired polydopamine (pDA) coating method. This technique allowed for a controlled release of silver ions, effectively prevented infections, and promoted bone mineralization [54]. These properties of electrospun scaffolds seem to remain consistent for various polymers and cell types [55,56,57].

#### 3.1.2. Stimulating Angiogenesis

Another notable attribute of electrospun scaffolds, especially pertinent in the realm of cardiac tissue engineering, is their capacity to promote angiogenesis. Current therapies may alleviate the symptoms of myocardial ischemia, but they lack the capability to restore necrotic myocardial tissue. However, cardiac tissue engineering approaches such as electrospinning may offer a new therapeutic solution to this deficiency.

Various animal experiments have demonstrated electrospun scaffolds’ efficacy in stimulating angiogenesis post-myocardial infarction. This is largely attributed to their capacity to efficiently carry epidermal factors, vasculogenic factors, and angiogenic factors, as well as molecules possessing antimicrobial and anti-inflammatory properties. Wang et al. highlighted the potential applications of bioactive ions and nanostructured biomaterials in cardiovascular tissue engineering by incorporating optimized concentrations of calcium silicate into aligned chitosan electrospun nanofibers. This resulted in composite cardiac patch scaffolds that heightened levels of cardiac and angiogenic markers, enhanced myofilament structures, and improved calcium signaling in neonatal rat cardiomyocytes [58]. Furthermore, electrospun scaffolds may facilitate the dispersion and promote the adhesion of epithelial cells and fibroblasts. Deepthi et al. aimed to create a tendon construct for flexor tendon regeneration by layering chitosan–collagen hydrogel on electrospun aligned poly (l-lactic acid) (PLLA) nanofibers and then coating it with alginate gel. They found that both uncoated chitosan–collagen/PLLA scaffolds and alginate gel-coated scaffolds demonstrated good cell proliferation [20].

The flexibility in the design and structure of electrospun scaffolds also enables them to serve as templates for developing new blood vessels, enhancing circulation, and promoting healing at specific anatomical sites. A study by Chung et al. showed that electrospun PLLA mat effectively facilitated sustained VEGF release and the delivery of cardiac stem cells, leading to enhanced angiogenesis and cardiomyogenesis in the setting of acute myocardial infarction, which could potentially improve myocardial regeneration [59]. A similar study by Spadaccio et al. demonstrated that a poly-l-lactide scaffold releasing granulocyte colony-stimulating factor effectively integrated into chronically infarcted myocardial tissue, promoted angiogenesis, reduced inflammation, induced ECM remodeling, and ultimately contributed to improved cardiac function and prevention of ventricular dilation in a rabbit model of chronic myocardial infarction [60].

#### 3.1.3. Improving the Mechanical Properties of Scaffolds

The heart, a complex physiological pump, is comprised of a variety of cells. To construct functional heart muscle that replicates the properties of native heart tissue, there is a need for scaffolds that exhibit specific electrophysiological and mechanical properties. The ideal biomaterial for cardiac tissue engineering should mirror the heart’s uniqueness. It should possess exceptional flexibility and elasticity and be capable of withstanding millions of contraction cycles, all while promoting the viability and differentiated characteristics of seeded cells.

Electrospinning offers a means to attain the desired biocompatibility, conductivity, and mechanical strength in various applications. For instance, Bertouli et al. employed core/shell electrospinning to create electroactive and biocompatible fibrous scaffolds using polyaniline (PAni) doped with dodecylbenzenesulfonic acid (DBSA) and blended with poly(lactic acid) (PLA) and PLA/poly(ethylene glycol) (PEG) mixtures. They demonstrated that incorporating PEG enhances the packing of PLA and PAni chains, allowing control over fiber thickness and improving electrical conductivity, thus favoring normal cardiomyocyte beating and contraction functions. Additionally, the biocompatibility of the fibers increased significantly when a shell coating of the PAni-containing system was incorporated via coaxial electrospinning, which slowed the release of PAni. The study concluded that these fibrous scaffolds, especially those with a shell coating, are suitable for cardiac tissue engineering applications, as demonstrated by morphological and functional studies with cardiac cells [61]. Ensuring electrical conductivity is crucial for achieving synchronized cardiomyocyte contractions in cardiac scaffolds, prompting the exploration of various fabrication methods [62].

Additionally, it is important to produce scaffolds with anisotropic mechanical properties similar to native myocardium. Native heart tissue possesses a unique arrangement of collagen fibers, which contributes to its mechanical behavior. This can be achieved by adjusting fabrication parameters to yield different fiber orientations. A study by Kai et al. showed that electrospun poly-glycerol sebacate fibers can be aligned in order to mimic this anisotropy. These nanofibers demonstrated anisotropic mechanical properties, with higher modulus and tensile strength along one direction, matching the contractile orientation of cardiomyocytes in the heart. Importantly, these mechanical properties were soft enough not to inhibit cardiac contraction while maintaining their integrity. This study also revealed that these nanofibrous scaffolds had a profound impact on cardiomyocyte alignment and orientation [63].

### 3.2. Electrospun Scaffolds for Heart Valve Replacement

#### 3.2.1. Challenges in Heart Valve Tissue Engineering

Heart valve replacement is a significant potential clinical application of tissue engineering endeavors. Current treatment options mainly involve valve replacement or repair. Valve replacement can involve mechanical or bioprosthetic allograft valves or, in rare cases, homograft (donor) valves. While these procedures save lives, they are not without limitations, such as the need for lifelong anticoagulation with mechanical valves and the risk of calcification or mechanical failure with allografts. Furthermore, mechanical replacements, allografts, and homografts lack the ability to grow, which poses a significant challenge for pediatric patients with congenital valvular disease. To tackle this problem, research into tissue-engineered heart valves has been ongoing for more than three decades as an alternative replacement approach [64].

However, there are several challenges with tissue engineering that must be solved before becoming a viable therapy for valvular disease. Fioretta et al. identified two major groups of challenges in the field of heart valve tissue engineering: clinical challenges and technical challenges. Clinically, tissue-engineered heart valves must prove their superiority over existing bioprosthetic valves for widespread clinical adoption. Current advancements in the durability and design of bioprosthetic valves, along with minimally invasive implantation techniques, continue to drastically increase the standard for clinical application. Tissue-engineered heart valves must also compete with commercially available decellularized homograft and xenograft valves currently in clinical trials [65].

Technical challenges include donor-to-donor variability in cell culture, optimization of the seeding processes, and achieving a synthetic matrix that replicates the unique functional characteristics and microstructural organization of native heart valves. Balancing collagen production and scaffold degradation is crucial for ensuring tissue-engineered heart valve functionality over time. Additionally, cell infiltration and the host response’s macrophage phenotype should be carefully considered. The variability of in vivo regeneration among patients, influenced by age and comorbidities, requires further investigation. Innovative in vitro technologies and a correct set of markers to monitor tissue remodeling and healing are essential for predicting clinical outcomes and understanding the differences between human and animal responses to biomaterials [65].

#### 3.2.2. Applications of Electrospinning in Valve Tissue Engineering

Electrospun polymeric scaffolds have the ability to address some of these technical challenges. The creation of scaffolds with ideal attributes, including strength, degradation rate, porosity, microstructure, shape, and size, is more controllable and reproducible in polymeric scaffolds. Polymeric scaffolds possess unique features, such as a high surface-to-volume ratio, small pore size within a highly porous structure, biodegradability, and favorable mechanical properties, making them highly appealing. Their advantages include biocompatibility, versatile chemistry, and significant biological properties, all of which are of great importance in the field of heart valve tissue engineering [66].

This concept was explored in a study by Jana and Lerman. They aimed to construct three-layered tissue-engineered heart valve leaflets that closely mimic native structure and function in rat models. Over three months, the scaffold material degraded during in vivo tissue engineering, and the tensile properties of the tissue constructs improved, becoming sufficient to withstand physiological pressure. As the scaffold material continued to degrade after heart valve replacement, complete tissue leaflet constructs with native properties were expected to develop. The mechanical characteristics of the scaffolds and tissue constructs were similar, indicating that the scaffolds’ anisotropic properties influenced the anisotropy of the resulting tissue. Additionally, the engineered tissue constructs displayed the presence of collagen, glycosaminoglycans, and elastin, mirroring the composition seen in native leaflets. The trilayered and oriented fibrous scaffolds designed in this study hold the potential to be valuable in the creation of scaffolds for effective heart valve replacements [67]

Recent studies have demonstrated that electrospinning can be used to facilitate the function of decellularized heart valves. A study by Wang et al. demonstrated that decellularized valves reinforced with electrospun scaffolds had significantly improved tensile strength and strain [68]. Given the inherent strength of electrospun scaffolds, these findings are not surprising. In fact, another group developed electrospun poly(carbonate urethane) (ES-PCU) mesh coated with a poly(ethylene glycol) diacrylate (PEGDA) hydrogel material for the creation of valvular leaflets. These bioengineer leaflets were able to demonstrate tensile properties well within the desired functional range for such a material [69]. Another potential application of electrospun scaffolds in the treatment of valvular disease is the work by stadelmann et al. This group was able to create an in vitro 3D model of calcific aortic valve disease by creating bi-layered cryogenic electrospun scaffolds that allowed for appropriate growth ingrowth of valvular cells [70]. All in all, the use of electrospinning technology in the treatment of valvular heart disease represents a promising new frontier of potential therapies.

### 3.3. Electrospun Patch for Infarct Repair

#### 3.3.1. Functionalizing the Patch for Controlled Drug Delivery

Controlled drug delivery seeks to enhance drug solubility and bioavailability or regulate the timing and location of drug release. Electrospun patches provide ideal conditions for controlled drug delivery due to their increased surface area-to-volume ratio, surface functionality adaptability, and superior mechanical performance, including stiffness and tensile strength, surpassing other known material forms. Moreover, they offer additional advantages conducive to creating an optimal drug delivery system, including high drug loading capacity, efficient drug encapsulation, the potential for concurrent delivery of multiple therapies, ease of use, and cost-effectiveness. It is worth noting that electrospinning can accommodate a wide range of drugs, both hydrophobic and hydrophilic, as well as larger biomolecules like proteins and DNA. These characteristics render electrospun patches remarkably versatile in potential applications and a promising method of drug delivery to the myocardium [5,57].

The versatility of the electrospinning process allows numerous methods for effectively introducing therapeutic agents into electrospun nanofibers. Zamani et al. identified the most popular methods of drug incorporation in electrospun scaffolds: blending, surface modification, coaxial electrospinning, and emulsion electrospinning [71]. Blending, the most common method, involves mixing the therapeutic agent directly with the polymer solution prior to electrospinning. This method is straightforward but requires careful consideration of the physicochemical properties of both the drug and the polymer to achieve efficient encapsulation. Matching the hydrophobic or hydrophilic properties of the drug with the corresponding polymer is crucial for successful encapsulation. Surface modification involves attaching or conjugating therapeutic agents to the surfaces of the electrospun nanofibers. By doing so, the drug remains on the fiber surface, allowing for controlled and sustained release. This method is particularly useful for addressing issues like burst release and short release times. However, it may not be suitable for drugs that require endocytosis or interaction with cell nuclei, as surface-bound molecules tend to have limited release. Coaxial electrospinning is a modified electrospinning technique that creates fibers with a core–shell structure. Here, the therapeutic agent is placed in the core, and it is electrospun simultaneously with a polymer solution forming the outer shell. This method offers several advantages, including sustained release, preservation of bioactivity, and efficiency. Lastly, emulsion electrospinning involves emulsifying the therapeutic agent (e.g., drug or protein) within a polymer solution. After electrospinning, the biomolecule is distributed either within the fibers or in a core–shell structure, depending on factors like the ratio of aqueous to the polymer solution. Unlike the blending technique, the drug and polymer are dissolved in appropriate solvents, eliminating the need for a common solvent. This allows for the use of various hydrophilic drugs and hydrophobic polymeric combinations and the preservation of bioactivity. However, the emulsion can potentially damage or degrade sensitive macromolecules, such as DNA [71].

#### 3.3.2. Integration of Patch with Native Tissue

The integration of electrospun drug-loaded patches with native tissue is a crucial aspect of their effectiveness in various biomedical applications, particularly in the context of tissue engineering for cardiac infarct repair. Many of the aforementioned characteristics of these patches are vital in accomplishing this goal. Their ability to mimic the mechanical properties of the native extracellular matrix, promote cell adhesion and proliferation, and promote angiogenesis gives these patches the unique potential to regenerate healthy cardiac tissue and restore cardiac function [59,63]. This was demonstrated in a study by Shafiq et al. in which they fabricated electrospun cardiac patches capable of releasing substance P and insulin-like growth factor-1C peptide, aiming to facilitate the regeneration of infarcted myocardium. The patches showed significant recruitment of bone marrow mesenchymal stem cells in vitro compared to controls. In murine MI models, the patches yielded better heart function, attenuated cardiac remodeling, and increased formation of blood vessels and capillaries compared to other groups [72].

### 3.4. Electrospinning and Drug Delivery in Heart Disease

#### 3.4.1. Incorporation of Therapeutic Agents into Electrospun Nanofibers

In recent years, cardiac patches have experienced a rise in popularity as a targeted method of delivering therapeutic compounds to the heart. This is particularly relevant in the context of coronary artery disease, myocardial infarction, and tissue repair. Regenerative treatments involving living cells, proteins, and genetic materials seek to change the course of unfavorable cardiovascular remodeling and encourage the creation of new cardiac tissue. However, successfully delivering these therapies to the heart has proven to be a challenging endeavor. Electrospun polymeric nanofibers represent a promising strategy for creating a controlled drug delivery system of the variety needed to surmount this barrier [3,5].

A study by Chung et al. demonstrated the potential effectiveness of these drug delivery systems. They developed an epicardial delivery system using a poly(l-lactic acid) (PLLA) mat to deliver vascular endothelial growth factor (VEGF) and cardiac stem cells to the infarcted myocardium. The VEGF-loaded PLLA mat, created through electrospinning, enabled sustained VEGF release over four weeks and promoted the migration and proliferation of endothelial cells and cardiac stem cells in vitro. Additionally, it positively affected the formation of capillary-like networks by endothelial cells. This study contributes to the increasing body of evidence supporting the potential utility of drug-loaded cardiac patches created through electrospinning in stimulating angiogenesis and cardiomyogenesis following acute myocardial infarction [59,73,74,75].

#### 3.4.2. Controlled Release Systems for Cardiac Drug Delivery

Controlled-release drug administration seeks to precisely control the duration of drug delivery. This approach prioritizes the predictability of drug release patterns. This shift offers substantial advantages, particularly in terms of patient adherence and the mitigation of potential side effects. In clinical cardiology, where many patients require lifelong drug therapy, adherence to treatment and the ability to tolerate a drug are critical considerations [76].

Traditionally, oral and transcutaneous controlled drug delivery systems have been employed to achieve these goals. However, these systems lack precision in targeting specific tissues, potentially reducing the drug’s therapeutic effectiveness and increasing the risk of systemic side effects. This challenge is particularly relevant in the context of cardiovascular disease, where lesions may be highly localized. Conversely, localized drug delivery using electrospun fibers can lead to reduced drug dosage requirements, minimizing systemic absorption and diminishing undesirable side effects. When compared to other drug carriers such as liposomes, hydrogels, and conventional nano/microspheres, electrospun nanofibers offer significantly improved drug encapsulation efficiency while also reducing the initial burst release through the careful selection of drug–polymer–solvent systems or electrospinning techniques [5,71,76,77].

The efficacy of drug-loaded electrospun scaffolds in promoting controlled drug release has been explored in recent studies. Kundrat et al. explored the electrospinning of poly(3-hydroxybutyrate) (PHB) to create different scaffold structures for drug incorporation, using levofloxacin as a model drug. The electrospun PHB scaffolds showed promise in entrapping and releasing a sufficient amount of levofloxacin to exhibit antimicrobial effectiveness. The study suggests that the electrospinning method used to create sponge-like scaffolds has the potential to develop an efficient and controllable drug release system. Similar studies also support the feasibility of these controlled drug delivery systems [78,79,80].

#### 3.4.3. Electrospun Nanofiber-Based Drug Delivery for Atherosclerosis Treatment

Electrospun nanofiber-based technology is of particular interest in the treatment of atherosclerosis. Because current non-specific therapies possess drawbacks, including limited distribution in the body, rapid elimination, and unwanted side effects, new strategies are required to enhance the treatment of this disease entity. There has been recent interest in the use of nanotechnology to help address some of these limitations [81].

In recent years, researchers have explored the therapeutic potential of electrospinning technology for addressing atherosclerosis, primarily through the development of stents coated in drug-eluting electrospun material. Studies in animal models have demonstrated that these stents may offer significant benefits over bare metal stents in the treatment of atherosclerosis. When investigating the effects of matrix elongation on the structure and drug release kinetics of polycaprolactone (PCL)-based electrospun paclitaxel (PTX)-enriched matrices, which are used as coatings for drug-eluting stents (DES), it was found that the arterial wall’s ability to retain and accumulate PTX results in prolonged drug release in rabbit models and could potentially allow for lower drug doses in the electrospun-produced coatings of stents [82]. A similar study on DES coated with an electrospun blend of polycaprolactone, human serum albumin, and paclitaxel found that they were less traumatic and induced less neointimal growth compared to bare metal stents in rabbit models [83]. Another study on a DES coated with a polycaprolactone (PCL) and polyurethane (PU) blending coaxial nanofiber showed that the drug release rate from the stent could be controlled by modifying the PTX loading ratio in the nanofiber’s core and shell in vitro. This suggests that the PCL/PU nanofiber-coated stent could also be adjustable to accommodate individual patient conditions related to blood vessel diseases [84].

#### 3.4.4. Alternative Bioengineered Patch Strategies

The concept of bioengineered patches for the treatment of cardiac disease is not limited to electrospun nanoscaffolds. Multiple dECM patches are either under investigation in clinical trials (ventrilGel and PeriCord) or have received approval (CorPatch) [85]. While dECM is an attractive patch material, it is limited by the fact that its hierarchal structure is determined by the native tissue it is harvested from [86]. Hydrogel patches represent another popular area of investigation for the treatment of cardiac disease. Hydrogels have the advantage of being able to incorporate either synthetic materials or dECM as part of their foundational matrix; however, by their very creation, the natural structure of their foundational material must be broken down and polymerized, thereby altering the crosslinking network that determines their overall hierarchal structure [87]. Recent studies have demonstrated improved functional cardiac outcomes in a rat MI model treated with an acellular hydrogel patch, and this may represent another viable bioengineering strategy for the treatment of ischemic heart disease [88].

## 4. Challenges and Future Perspectives

Drug delivery systems are developed to optimize therapeutic outcomes while minimizing potential toxicity concerns. The rapid advancements in delivery technologies have spurred the creation of various materials with improved properties, such as smaller size, increased permeability and solubility, stability, and specific site targeting [89]. Electrospinning has emerged as one of the most effective methods for synthesizing these materials and has garnered significant attention due to its versatility and accessibility in production methods and applications. The flexibility of electrospinning technology allowed the production of a rich variety of fiber morphologies, which greatly expedited the advancement in the fields of pharmaceutical drug delivery, biomedical applications, and tissue engineering [90,91].

Significant progress in electrospinning technology has been made from the late 20th century to the early 21st century, largely spurred by the increasing need for nanotechnology. Electrospinning is distinguished by its simplicity, high efficiency, cost-effectiveness, and reproducibility, as it offers controllable fiber deposition and predefining pattern construction. Given its capability to generate products with tailored compositions, dimensions, and structures, coupled with the rising demand for nanofibers in healthcare industries, this section explores the challenges and future prospects of electrospinning technology [90,92].

### 4.1. Scalability and Commercialization of Electrospinning Technology

As noted earlier, electrospinning allows the incorporation of a wide range of drugs within the fibers, thereby improving bioavailability, dissolution, and controlled release. For instance, the electrospun fibers offer increased surface area and porosity, allowing for improved drug solubility and dissolution rates. Additionally, the controlled release of drugs from electrospun fibers is achievable due to the tunable parameters of electrospinning, such as polymer concentration and processing conditions, which enables the modulation of drug release kinetics to meet specific therapeutic requirements. Moreover, electrospinning offers an energy-efficient and cost-effective alternative to the commonly used freeze-drying or spray-drying methods. Nevertheless, the majority of the existing publications utilize electrospinning with relatively low productivity, typically less than 1 g/h [93], and this limited production rate in laboratory-scale electrospinning has impeded its adoption in industrial contexts. Numerous efforts have been made to scale up this technology to achieve higher production rates by changing nozzle and speed parameters or utilizing free-surface methods [93,94].

The most straightforward means to enhance scalability is multi-needle electrospinning, with the arrangement and spacing of two or more needles allowing for various configurations. However, the mutual interactions of the electric fields between adjacent needles pose challenges for this approach. Such interactions can result in repulsive forces between the liquid jets, potentially leading to increased variability in fiber quality [93,95]. Theron et al. conducted experiments on two multi-jet electrospinning setups (3 × 3 matrix and 7 × 1 or 9 × 1 linear array, with inter-nozzle distance of 1–5 cm) to investigate the interactions between the jets. They demonstrated that the trajectories of charged jets are affected by both self-induced Coulomb repulsions and an externally supplied electric field, as well as the mutual Coulomb repulsions of the individual charged jets themselves. Consequently, the jets at the periphery of a configuration displayed a significant outward curvature, while the inner jets were compressed along the line where the rotating nozzles were situated. This prompted the authors to recommend maintaining an inter-nozzle gap of approximately 1 cm to ensure a stable and uniform nanofiber formation [96]. Moreover, an improper arrangement of the collectors or downward spinning of the jets may lead to the bead production or potentially cause disruptions in the fiber-forming process [91]. The electro-blowing method, wherein an additional airflow facilitates solvent evaporation, provides much higher production rates (42.5–56.7 g/h). However, the airflow during the electro-blowing process may result in a higher occurrence of beads and droplets within the fibers. Finally, it is important to note a key limitation inherent to all nozzle methods: the potential for spinneret clogging with increased feeding rates [93,97]. Thus, fine-tuning the process parameters, including feeding rate and applied voltages, becomes necessary, and this process of optimization presents further challenges as only some of the parameters can be easily varied.

The free-surface electrospinning method circumvents the clogging issues seen with nozzle methods. In this method, the fibers are produced directly from an open surface, replacing the need for nozzles. This approach was first introduced in a patent featuring a rotating charged cylindrical electrode submerged in the solution as the initial surface for fiber generation, which was later commercialized as *NanospiderTM* by Elmarco [98]. In a subsequent iteration, the cylindrical electrode was replaced with a wire covered by the solution through a continuous feeding system. Continuously, various other spinneret types, including beaded-chain, spiral coil, balls, and bubbles, have been introduced to enhance the productivity of the free-surface method, producing up to a hundredfold increase to 450 g/h. Nevertheless, these methods also come with certain challenges, including the rapid evaporation of volatile solvents commonly used to dissolve poorly water-soluble drugs. This rapid evaporation results in fluctuations in the concentration within the liquid film, reducing the quality of the product [91,93].

Unfortunately, there have been limited notable developments in the creation of electrospinning devices specifically designed for industrial scale. *NanospiderTM* is still the only commercially available device utilized in pharmaceutical applications despite the ongoing advancements regarding scaling up instruments and products [93,99]. Hence, industrial translation requires more support and progress, especially from corporations, as the majority of existing research in this field originates from academic sources.

Recently, rotary jet spinning (RJS) has emerged as a potential solution to the challenges posed by electrospinning, providing higher production rates, low cost, and environmental friendliness [100,101,102]. The RJS system comprises a reservoir with two side wall orifices attached to the shaft of a motor with controllable rotation speed. As the reservoir rotates on its axis, it expels a polymeric jet through the nozzles by centrifugal force. The solvent evaporates to solidify and shrink the polymer jet as it follows a spiral path, forming thin fibers. The nozzle geometry, rotation speed, and polymer solution properties can be modified to control the polymer fiber diameter and porosity. For instance, highly volatile solvents will result in thicker fibers, as their rapid evaporation potentiates rapid solidification, hindering the stretching of the jet [103]. Past studies have compared direct nanofiber production between the two methods. For example, Rogalski et al. compared polyamide 6 (PA6) nanofiber production in terms of fiber diameter, variation, and crystallinity. They showed solution-concentration-dependent changes in fiber diameter, where electrospun fibers had smaller diameters up to a polymer in solution concentration of 22.5 wt%, after which point RJS produced a smaller diameter. The fiber diameter variation was greater in RJS, ranging from 350 ± 180 nm to 500 ± 250 nm, compared to electrospun fibers, which ranged from 40 ± 10 nm to 1250 ± 150 nm. The crystallinity of the fibers was comparable in both methods, 33% and 30% for electrospun and rotary jet-spun fibers, respectively [101]. Another study by Machado-Paula et al. investigated morphology and diameter changes, as well as the antifouling effects of the fibers produced from the two techniques. Micrograph images showed that RJS fibers presented higher roughness independent of concentration compared to the smooth fibers obtained by electrospinning. Colony-forming unit assays, along with SEM image analysis, showed that RJS fibers decreased bacterial colonization for both Gram-positive (*S. epidermidis* and *S. aureus*) and Gram-negative bacteria (*P. aeruginosa*) compared to electrospun fibers [102]. More recently, researchers have focused on exploring the broad applicability of RJS. Unlike electrospinning, which requires high-voltage electric fields and a conductive solution, RJS uses high-speed rotation for producing aligned fibers, requiring a low boiling point of the solvent [100]. Without electric field interference, RJS allows fiber production from nonvolatile solvents and from polymers with charged groups. A newer method called immersion rotary jet spinning (iRJS), derived from RJS, further eliminated the need for volatile carrier solvents by using a vortex-controlled precipitation bath for fiber solidification. As a proof of concept, Gonzalez et al. spun nanofibers that cannot be readily formed using conventional electrospinning techniques, such as poly (para-phenylene terephthalamide) (PPTA), nylon, DNA, and alginate. Furthermore, the team produced alginate–gelatin nanofiber scaffolds using this method and cultured them with C2C12 myoblasts. The scaffolds, where the diameter depended on the solution concentration, showed elastic modulus values ranging from 5 to 60 kPa, comparable to native skeletal muscle, and cell attachment, proliferation, and differentiation were maintained for up to 2 months [104]. Together, these results suggest an attractive and reliable method for fiber production, addressing the limitations of traditional electrospinning methods. However, given that it is a relatively new technique, more understanding is needed before it could be widely implemented.

### 4.2. Improving Mechanical Strength and Degradation Rate

Several key parameters, including processing parameters, solution constituents, environmental conditions, and the collector design, determine the “processing window” or “optimum range”, beyond which electrospinning is not possible [105,106]. The key processing parameters are the applied electric field, needle-to-collector distance, solution feed rate, and needle diameter. The stretching force, directly proportional to the applied electric field, causes a reduction in fiber diameter with the increase in the applied potential difference between the needle and collector. Upon exceeding the optimum range, especially in elevated applied voltage or increased solution feed rate, bead formation or ribbon-like defective morphology is noted. In terms of solution constituents, appropriate solvent selection, polymer concentration, and solution conductivity play critical roles. The choice of a solvent depends on the compatibility with the polymer and its boiling point, which in turn determines its volatility. A solvent with a modest boiling point would evaporate quickly before the fibers are deposited on the collector, and its low volatility would prevent the needle from being clogged [107]. Polymer concentration directly influences solution viscosity, with high concentrations resulting in larger diameter bead-free fibers due to increased polymer chain entanglement. Conversely, reducing polymer concentration below an optimum level leads to low viscosity, causing polymer jet fragmentation and the formation of beaded, discontinuous fibers. Finally, the conductivity of the solution affects the surface charge density, which facilitates the electrostatic contact between the droplet at the needle tip and the collector. This leads to the creation of a Taylor cone, which marks the start of the electrospinning process. Increased solution conductivity also enhances the repulsive contact between fiber bends, amplifying the whipping action and producing fiber stretching and ultimately thinning [108]. Environmental factors like relative humidity and ambient temperature influence fiber morphology, and collector configuration impacts fiber alignment, diameter, and mechanical properties. Successful electrospinning relies on careful calibration of these parameters to ensure defect-free, continuous fibers with desirable properties [91].

The resulting fibers, however, often exhibit limited mechanical strength, described as a “cotton-like” state, with little inter-fiber cohesion and structural integrity. Some solution constituents, such as collagen, exhibit reduced strength, primarily due to their fragile composition and porous structure. The scaffolds developed by Barber et al. in 2013 and Petrigliano et al. in 2015 were able to promote cell adhesion and facilitate cell infiltration with their laser-cut pores, respectively, but both approaches have shown mechanical strength that falls short of that exhibited by native ligaments [109,110]. Even materials with excellent mechanical properties often experience decrements when applied to electrospun membranes [111]. Additionally, during processing, the electric field forces that stretch the fibers lead to the poor orientation of macromolecule chains within the fibers, failing to crystallize them completely [111]. The variations in the orientation and configuration of the fibers result in a lack of uniformity in the direction and timing during the process of stress.

Oftentimes, these “cotton-like” fibers are difficult to handle and thus unsuitable for practical applications, often requiring post-processing treatments to improve their structural integrity and mechanical properties [105,106]. The post-processing treatment strategies encompass a combination of chemical and physical methods, such as annealing, post-stretching, twisting, solvent steam, and cross-linking. The impact of post-fabrication treatments is evaluated to confirm minimal alterations to fiber morphology, diameter, porosity, and pore tortuosity [105]. Interestingly, Abhari et al. observed that annealing temperature can be adjusted to modulate the mechanical properties and degradation rate of electrospun materials. They found that annealing at 65 °C for three hours significantly reduced degradation while only slightly enhancing the initial tensile strength (9 ± 2%). Conversely, annealing at 75 °C improved the initial tensile strength of the filament (17 ± 6%). The samples treated above 75 °C performed comparably or mechanically worse than those not annealed. Taken together, these observations suggested a valuable means to tailor the characteristics of electrospun materials without altering the polymer’s chemistry [112]. Another study demonstrated that the fabrication of nanofiber mats from Poly(m-phenylene isophthalamide) (PMIA) through electrospinning, followed by hot stretching along the fiber axis, enhanced their mechanical properties. Fiber alignment and diameter, as well as the crystallinity and glass transition temperature, were measured with scanning electron microscopy, X-ray diffraction, and differential scanning calorimetry, which showed that hot stretching led to improved alignment and enhanced crystallinity and glass transition temperature [113]. Roman et al., Salimbeigi et al., and Yang et al. performed experiments to show similar effects of twisting, solvent steam, and cross-linking on the mechanical properties of the electrospun fibers, respectively [114,115,116]. Other approaches to enhancing the strength of nanofibers include adding suitable filling to the raw polymer materials or altering the parameters of the electrospinning process to increase chain orientation and crystal in the fiber [111]. Spearman et al. built a more robust nanofiber membrane combining low-strength polycaprolactone (PCL) and high-strength polyglycolide (PGA). The mechanical properties were assessed through three-point bend testing, and the results demonstrated that the composite fiber led to increased tensile yield strength and Young’s modulus compared to the pure fibers alone. Further analysis suggested that the addition of polyglycolide increased the crystallization percentage of the fibers [117].

However, challenges still persist in finding the optimal method to meet the required mechanical strength. At present, a combination of different procedures is commonly adopted to increase the effectiveness: for example, the combination of stretching and annealing treatments has been shown to yield nanofibers with exceptional strength and fineness through reaction between highly organized arrangements of multiple fibers [111,118]. In fact, Liao et al. showed that the collection of electrospun yarn and poly-acrylonitrile-co-methyl acrylate fibers annealed under tension, aligning the small fibers, and cross-linked through PEG-BA, demonstrated a toughness of 137 ± 21 joules per gram and a tensile strength of 1236 ± 40 megapascals. These properties were comparable to dragline spider silk, which is known for its combination of strength and toughness [118]. In the future, it is imperative to conduct thorough research to gain a deeper understanding of electrospinning principles and the fiber-forming process in order to unlock its potential for innovative applications.

Despite the promise inherent in biodegradable polymers, there is still a long way to go to translate them into clinical and commercial applications. Aligning the degradation properties of electrospun fiber scaffolds with tissue repair rates remains an extremely challenging task. Some studies have suggested optimizing the combinations of substrate materials, introducing additional components, or modifying the scaffolds as a way to address the issue. For example, Spagnuolo and Liu effectively controlled the degradation of the electrospun scaffold by blending two biodegradable L-tyrosine-based polyurethane materials that had different degradation rates [119]. Similarly, Thao et al. increased the proportion of the slower degradation-, hydrophobic materials poly(ε-caprolactone-*ran*-l-lactide) (PCLA) to slow the drug release rate [120]. Other studies have shown that adding a catalyst or nano-hydroxyapatite (nHA) into electrospun poly(L-lactide) (PLLA) or adjusting the hydrophobicity of a polymer changed the degradation rate [121,122,123,124]. Finally, post-treatment through radiation or ultraviolet rays (UV) may change the degradation rate of the polymeric scaffold [119]. Adjustment of the implanted scaffold materials in response to tissue healing is needed using real-time imaging technologies, which is time-consuming and expensive, while a lack of understanding of pharmacokinetics once drugs enter the body presents an additional challenge in achieving optimal biodegradability.

### 4.3. Multifunctional Electrospun Materials for Personalized Medicine

Electrospun materials have found applications in medicine and healthcare, leveraging the microscopic and nanoscale characteristics of the electrospun fibers to deliver superior performance over conventionally manufactured materials. With their relatively high porosity and large surface-to-volume ratio, electrospun nanofibers can be applied to a wide range of medical uses, including tissue engineering, regenerative medicine, drug delivery, biosensors, and diagnostics [125].

Tissue engineering and regenerative medicine focus on the regeneration of damaged tissue, where cells are incorporated into porous scaffolds made of biomaterials and guide the growth of new tissue. Electrospinning has been extensively studied for the production of nanofibrous scaffolds, demonstrating promising potential in replicating the natural extracellular matrix (ECM) composition and architecture [125]. Electrospun fibers can aggregate and form a network that offers adjustable porosity, pore size, fiber diameter, and orientation, ensuring structural integrity and compatibility with native ECM. Recent investigations have shown that combining multiple approaches, including topographical and biomedical cues, can be used to better guide stem cell differentiation and tissue regeneration. For example, incorporating bioactive agents can introduce biological cues, while careful material selection and control of fiber chemistry, thickness, and porosity can provide electrochemical stimulation. The three-dimensional network, with fiber orientation varied across tissues, allows replication of complex and anisotropic structures as well [125,126]. These findings may provide an avenue to address challenges that have arisen from the mechanical mismatch between electrospun scaffolds and native ECM, difficulty replicating tissue-to-tissue interfaces, or providing specific functionality such as electrochemical stimulation for neural and cardiac tissue [125].

Electrospun materials also make an ideal carrier for drug delivery, given their large surface-to-volume ratio, allowing high drug loading capacity and efficiency. In addition, the combination of a variety of polymers accessible for electrospinning along with process parameters allows the development of complex controlled release systems. Drug-eluting electrospun fibers have been developed for use in a wide range of applications, including topical, transdermal, implantable, injectable, and oral delivery [125,127]. While various drug-loading methods yield distinct interactions between the drugs and nanofibers, subsequently affecting the kinetics of drug release, a few trends exist [127]. First, it is important to formulate the fiber with compatible drugs and carrier polymers to preserve the bioactivity of the drugs. Furthermore, the composites of electrospun fiber allow for the desired release profile, either through promoting tissue integration or achieving the desired release rate. For example, the initial burst of drug release can be advantageous for anti-inflammatory effects in some disease contexts, while controlled and sustained drug release is more desirable in more complex scenarios [125,127]. Finally, the combination of electrospun fibers with other drug delivery systems may expand the breadth of potential applications. One such example is the construction of a “smart” drug delivery system, which provides target-specific, triggerable controlled release, mitigating the potential damage of chemotherapy to surrounding tissue. For instance, one group successfully synthesized a copper silicate hollow microsphere (CSO HMSs)-incorporated electrospun scaffold for the treatment of melanoma. The Cu-based chalcogenides absorb near-IR light intensely, making them effective photothermal agents, while prior evidence has shown that Cu ions aided wound healing. Thus, the researchers designed CSO-HMSs to act as drug-loaded photothermal agents to provide localized photothermal therapy. The CSO HMSs demonstrated a drug-loading efficiency of 26.9% with Tranmetinib, a chemotherapy drug, while exhibiting a mass-dependent photothermal effect, reaching a plateau temperature of 50 °C at a power density of 0.65 W/cm^2^. Both the in vitro results and in vivo results supported the anti-tumor efficacy of this therapy. In vitro, the treated group exhibited a substantial decrease in cell viability, reaching as low as 0.1% in the cell viability assays after three rounds of laser irradiation. Similarly, in vivo experiments showed that the treated group had extensive damage to the nuclei of tumor cells, as evidenced by hematoxylin and eosin staining; Ki67 antibody immunohistochemical staining additionally confirmed a substantial reduction in tumor cell proliferation [128,129]. Taken together, it is clear that future opportunities lie in existing and emerging electrospinning technologies to achieve the multifunctional applications of electrospun materials.

### 4.4. Integration of Electrospun Scaffolds with Cardiac Cells and Tissues

**Figure 4:**
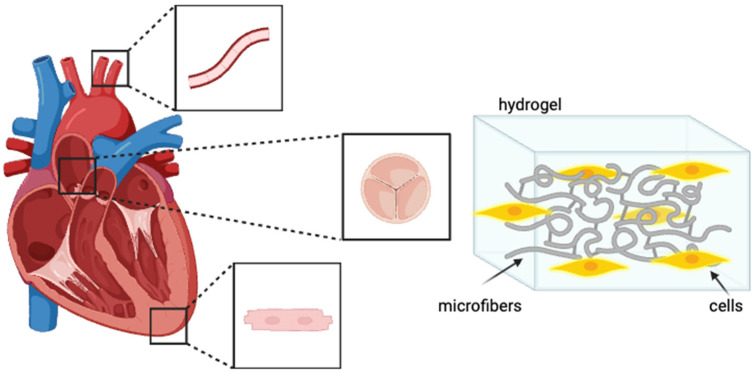
Electrospun nanofiber scaffolds have demonstrated promise in the treatment of a number of cardiac pathologies. Specifically, there have been recent developments demonstrating this technology’s potential for the treatment of atherosclerosis, valvular heart disease, and ischemic heart disease.

Cardiovascular disease (CVD) encompasses various conditions, including coronary artery disease, valvular heart disease, myocarditis, cardiomyopathy, and aortic aneurysm, with each arising after a distinct pathophysiologic process. The heart muscle is composed of electro-active tissues, and the regenerative potential of cardiomyocytes is relatively limited, resulting in a restricted capacity for self-repair [125,130]. Consequently, the challenge of repairing cardiomyocytes has persisted over the years up to the present. Recent advances in creating functional heart tissue, however, have accelerated CVD therapy, and electrospun materials provide an effective conductive and inducer scaffold suitable for platforms for myocardial and cardiac endothelial cells [125,130] (Figure 4).

Ensuring sufficient mechanical strength in cardiac tissue is paramount for maintaining the myocardium’s ability to contract and relax. The interwoven nature of the myocardium, with the coiled structure of perimysial fibers, poses additional challenges in replicating the organization and mechanical capability of cardiac tissue. Accounting for the complex nature of the cardiac tissues, Liu et al. developed scaffolds featuring a honeycomb pattern, and cardiomyocytes cultured on these patterned scaffolds exhibited heart rates comparable to those of neonatal or adult rats [125,131]. Moreover, Wang et al. further developed a conductive electrospun nanofibrous scaffold to mimic the electrochemical cues of the myocardium [132]. They have shown that the nanofiber sheets made of polylactide/polyaniline (PLA/PANI) created a conductive and biocompatible microenvironment for cardiac muscle cell (CM) viability, maturation, and synchronized beating. The increased myotube number and maturation index also observed indicated effective differentiation of H9c2 cells, along with improved cell–cell contacts. Immunofluorescence staining further revealed that PLA/PANI conductive sheets exhibited well-developed networks of sarcomeres and gap junctions of CMs, in contrast to PLA nanofibrous sheets alone. Moreover, spontaneous beating and the myocardial contraction pattern were sustained for up to 21 days of culture.

The 3D electrospun scaffold allows the orientation of nanofibers and the pattern of each layer to be individually modified, better mimicking the coiled perimysial fibers of the heart wall. Findings from Fleischer et al. demonstrated that a 3D spring-like fiber scaffold improved mechanical properties, including elasticity and extensibility, and improved ex-vivo function of cardiac cells, as evidenced by significantly stronger contraction force, higher beating rate, and lower excitation thresholds when compared to straight fiber scaffolds [132]. These results suggest potential materials for 3D bio-actuators and proportionally great promise in the field of cardiac engineering.

Electrospun nanofiber also offers a novel avenue for treating valvular disease by mitigating the risk of degradation, calcification, and the complex post-processing steps from the conventional animal valve approach. Nafiseh et al. developed tri-layered elastic scaffolds with adjustable anisotropic mechanical characteristics using microfabricated PGS and fibrous PGS/PCL electrospun sheets. They found that the engineered materials facilitated the growth of valvular mesenchymal stem cells and promoted the development of heart valve ECM. Their findings are especially useful in pediatric patients who require multiple surgeries over time for the replacement of non-viable prosthetic valves [133]. Jana and her team also employed electrospinning methods to fabricate three-layer natural valves using nanofibrous material. They employed in vivo tissue engineering to develop an autologous heart valve, where the nanofibrous substrate was inserted into the enclosed tri-leaflet-shaped chamber and surgically implanted in a rat model. After two months, the tissue construct was analyzed biologically, morphologically, and mechanically. Immunohistochemical staining showed the presence of three layers, and gene expression analysis revealed the presence of different ECM components, including collagen, glycosaminoglycans, and elastin, comparable to that in a native leaflet quantitatively [99].

While studies have successfully explored the biocompatibility and mechanical properties of the scaffolds, growing evidence suggests the effects of the dynamic mechanical environment on the development and differentiation of new tissues. Izadpanah et al. demonstrated cardiomyocyte differentiation from bone marrow-derived mesenchymal stem cells in both a static two-dimensional (2D) and a microfluidic three-dimensional (3D) environment. The results of real-time PCR and ICC analysis suggested that the expression of cardiac genes was significantly improved in the microfluidic cell culture that used both 5-Azacytidine (5-Aza) and a shear stress of 1 Pascal compared to that in the static 2D environment. Interestingly, no change was observed in the 5-Aza-only or shear stress-only groups [134]. A later study by Ravishankar et al. assessed the role of cyclic mechanical strain on endothelial progenitor cells’ (EPCs) differentiation. When EPC-seeded 3D anisotropic composites that mimic the aortic valve tissue matrix compositions were exposed to cyclic mechanical strain (at a rate of 1 Hz at 15% strain), they showed an improved potential to be differentiated into mesenchymal-like cells. Immunostaining and cellular analysis both showed reduced expression or uptake of epithelial markers and increased smooth muscle cell markers when subjected to mechanical stimulation [135]. Taken together, both studies highlight the importance of conducting mechanobiological testing in future studies.

## 5. Comparative Analysis with Other Tissue Engineering Approaches

Bio-fabrication approaches utilize manufacturing techniques, such as electrospinning, decellularization, and three-dimensional (3D) bioprinting, to organize biomaterials in a structured and hierarchical manner, integrating one or multiple cell types and molecules. Other techniques involve hydrogel-based tissue engineering. The ultimate aim is to create complex structures that mimic the properties and functions of natural tissues and organs.

### 5.1. Electrospinning vs. 3D Printing in Cardiac Tissue Engineering

Extrusion-based 3D printing is a popular method in tissue engineering, as it permits the fabrication of structures from both hard and soft materials. These structures provide a supportive framework for cells to adhere, proliferate, and generate new tissues, eventually resulting in the precise deposition of biomaterials. This precision allows for a more accurate replication of the natural architecture of organs and tissues. However, a significant limitation of extrusion-based 3D printing is poor resolution below a few microns, while important constituents of ECM are submicrons in size. Currently, it is challenging to add different scale lengths within the same structure, thereby reducing the structure’s biological applicability [136,137].

Electrospinning overcomes the major limitation of 3D printing by offering the opportunity to incorporate the entire range of the ECM from micro to submicron to nanoscale fibers. However, the traditional nanofiber scaffolds are mostly two-dimensional, and these nonexpanded scaffolds hinder cell infiltration, migration, and differentiation due to high density and small pore sizes. Moreover, the low mechanical strength of the scaffolds and uncontrollable shape also fail to create an environment resembling natural conditions [137,138].

Recently, the integration of 3D printing and electrospinning has been explored. A 3D network featuring both the printed and electrospun micro- and nanofibers presents a viable strategy that yields the benefits of both techniques, producing materials with high porosities, controllable shapes, adequate mechanical strength, and ECM-like properties [138]. Furthermore, cell behavior may also be altered by tailoring mesh topographical characteristics, such as fiber diameter, distribution, alignment, and porosity [136]. A combination of these two techniques can, in short, produce ideal biomaterials composed of microarchitectures that resemble the native extracellular matrix.

### 5.2. Electrospinning vs. Decellularized Scaffolds for Heart Regeneration

The use of decellularized extracellular matrix (dECM) has also gained significant attention as an attractive scaffold for regenerative medicine. dECM therapies entail harvesting tissue from either a donor or host, treating the tissue with detergents to remove nucleic acids and unwanted donor material that may trigger an undesirable immune reaction, and finally transforming the tissue into a suitable format for transplantation. With proper decellularization, dECM can retain components of the natural cell environment, including the biochemical and physical signals that promote cell recruitment, proliferation, and differentiation [139,140]. Moreover, dECM carries a lower risk of eliciting an immune response compared to transplanted tissue, as the majority of cellular DNA is removed. Finally, dECM can also be lyophilized and stored in powder, which can then be resuspended at varying concentrations. In fact, one group using blended dECM powder with poly (L-lactide-co caprolactone) created electrospun vascular grafts and was able to demonstrate improved cell adhesion and infiltration [141].

Nevertheless, decellularization also presents challenges, including optimizing the cellular material removal process to minimize ECM damage and scaling such materials to clinically relevant shapes and sizes [139,142].

**Figure 5:**
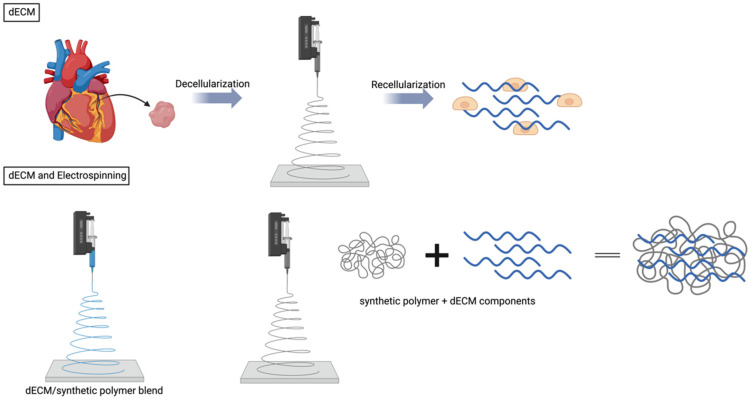
Schematic diagram of the two methods of incorporating decellularized extracellular matrix (dECM) into electrospun scaffolds, direct electrospinning of dECM materials, and post-electrospinning combination of dECM elements with scaffolds.

While a wide range of different scaffolding materials is available for electrospinning, the process is unable to fully replicate the native microenvironment and structure achieved with dECM. As a result, recent attempts have been made to achieve electrospun dECM scaffolds that can provide better control over the physiochemical properties of dECM while maintaining native ECM components [139,142]. Two approaches have been reported in the literature: adding components of dECM after the electrospinning process or direct electrospinning of dECM components. Maseali et al. utilized decellularization and processing techniques on human nasal septum cartilage constructs, which were then employed for the biofunctionalization of electrospun poly(hydroxyalkanoate) scaffolds [143]. On the other hand, Schoen et al. and Young et al. presented an alternative method whereby decellularized porcine cardiac tissue was blended with poly(ethylene oxide) or PLLA before electrospinning [144] (Figure 5). Regardless of the method, however, the combination of electrospun nanofiber scaffolds with dECM allowed the products to maintain their tissue-specific phenotype and produced better adhesion, proliferation, and differentiation, suggesting improved recreation of the tissue-specific microenvironment.

## 6. Limitations

It is worth noting that this review is limited to the discussion of electrospun nanoscaffolds. The problem of heart disease has spurred a myriad of bioengineering approaches, and many hold a great deal of promise. Advances in the fields of biocompatible 3D printing, injectable dECM, and stem cell-embedded implantable matrixes are not covered in this review. Many of these technologies may be used in conjunction with electrospinning, such as the work by magaz et al., who have demonstrated the ability to create scaffolds with conductive properties [145]. At the same time, other technologies represent giant leaps forward in the treatment of cardiac pathology independent of electrospun patches, such as the work by Noor N et al., who were able to create a 3D-printed heart [146]. While these and other topics represent important advances in potential therapies for this recalcitrant disease, it would not be possible to provide the reader with the appropriate level of information in the confines of this review. Rather, the authors hope to be able to provide the readers with a current update on electrospinning with our manuscript.

## 7. Conclusions

Despite the toll heart disease takes on the healthcare system worldwide, there remains a paucity of effective treatment modalities. Electrospinning represents a key bioengineering technique that, when leveraged correctly, possesses the potential to alter current treatment paradigms.

Electrospinning allows for a highly customizable substrate that mimics the functionality of a native extracellular matrix. This is of substantial value in ischemic heart disease, where morphological changes dictate functional decline post-infarct. Furthermore, electrospinning offers the ability to perform targeted drug delivery. Given the difficulties of achieving therapeutic drug levels in ischemic tissue due to poor blood flow, electrospun scaffolds represent a tremendous opportunity to deliver novel therapies both of a structural and of a pharmacological nature. Electrospinning has already demonstrated tremendous potential in valvular heart disease as a potential fabrication methodology for bioengineered heart valves. Furthermore, localized drug delivery has been demonstrated in the integration of this technology in stents for the treatment of atherosclerotic disease.

Despite these myriad advantages, there remain challenges in using electrospun biomaterials for large-scale therapeutic applications. In particular, the production rate using this technology, even with multi-needle electrospinning or electroblowing, is limited. Despite this limitation, the potential applications of this process for the treatment of heart disease should not be overlooked. Given its low cost of production and high degree of customization, electrospinning merits further investigation as a potential therapy. While there is much literature regarding the use of electrospun scaffolds in this disease process, the scientific community should continue to seek out the ideal combination of scaffold properties and drug delivery for the treatment of ischemic heart disease. Furthermore, there is a lack of large animal and human clinical studies that use electrospun scaffolds. This gap in knowledge will have to be addressed before the true therapeutic potential of this technology is known. Overall, electrospinning represents an exciting potential breakthrough in what has been an intransigent disease.

## Figures and Tables

**Table 1:** Summary of solution, process, and ambient parameters that alter nanofiber morphology.

Parameter	Effect of Fiber Morphology	References
Solution Parameters		
Concentration	Fiber diameter increases with polymer solution concentration. Beads form at low viscosity, and microribbons form at extremely high viscosity	[4,8,10,11,12,13]
Polymer Molecular Weight	Fiber diameter increases with molecular weight	[14]
Surface Tension	Occurrence of beads decreases with a decrease in surface tension	[4,8]
Conductivity	Occurrence of beads decreases with a decrease in conductivity	[15]
Process Parameters		
Flow Rate	Increasing flow rates are associated with an increase in fiber diameter	[4,8,9,16]
Voltage	Relationship may vary based on polymer formulation	[8,16,17]
Separation Distance	Beads form at large distances	[4,8]
Ambient Parameters		
Temperature	Increasing temperatures result in a decrease in fiber diameter	[18]
Humidity	High relative humidity causes varied fiber diameters and morphologies	[19]

**Table 2:** Nanofiber scaffold characteristics based on polymer solution and their current biomedical applications.

Material	Characteristics	Fiber Diameter * (nm)	Biomedical Applications	References
Natural Polymers				
Chitosan	Biodegradable Biocompatible Non-toxic Hydrophilic Hemostatic properties Anti-bacterial and anti-fungal properties	50–450	Tissue engineering scaffolds Wound dressings Sutures	[25,26,27,28]
Gelatin	Biocompatible Hydrophilic Biodegradable (slow) Promotes cell adhesion Poor electrospinnability Poor mechanical strength Typically blended with other polymers or modified to enhance properties and improve electrospinnability	100–340	Drug delivery systems Wound dressing Bone regeneration Tissue engineering scaffolds	[29,30,31,32,33]
Collagen	Biocompatible Biodegradable (slow) Poor thermal stability Promotes cell adhesion and proliferation Non-toxic, bad solvent stability Low mechanical strength	100–1200	Skin regeneration Wound dressing	[29,34,35,36,37]
Alginate	Biocompatible Biodegradable Low toxicity Antimicrobial High ion absorption Poor cell adhesion Poor electrospinnability Typically blended with other polymers or cosolvents to enhance properties and improve electrospinnability	120–300	Wound dressing Tissue engineering scaffolds Cancer therapy Delivery systems	[29,38,39,40,41,42]
Synthetic Polymers				
Polylactide (PLA)	Biocompatible Biodegradable (slow) Hydrophobic High mechanical strength Processable	360–430	Drug delivery Implants (stents) Sutures Tissue engineering scaffolds	[29,43,44]
Poly(lactic-co-glycolic acid) (PLGA)	Biocompatible Biodegradable (controllable) Tunable mechanical properties Non-toxic Poor cell adhesion	100–600	Drug delivery Tissue engineering scaffolds	[45,46]
Polycaprolactone (PCL)	Biocompatible Biodegradable Excellent mechanical strength Poor cell adhesion	320–1550	Wound dressings Drug delivery Tissue engineering scaffolds	[29,47,48]
Polyurethane (PU)	Biocompatible Variable biodegradability Hydrophobic Excellent mechanical strength Thermoplastic	456–1043	Medical devices Implants Drug delivery	[29,49,50,51,52]

* Plain electrospun nanofibers made of pure material.

## Data Availability

No new data were created or analyzed in this study. Data sharing is not applicable to this article.
